# Stretchable, Tough, and Luminescent Perovskite Molecular Ferroelectric Composite: Empowering Reliable Self‐Powered Motion Sensing in High Humidity and Subzero Temperatures

**DOI:** 10.1002/advs.76643

**Published:** 2026-07-20

**Authors:** Shuangqing Li, Zhe‐Kun Xu, Zheng Xing, Jin‐Wang Liu, Yong‐Quan Wu, Peng‐Fei Li, Zhong‐Xia Wang

**Affiliations:** ^1^ Center for Crystalline Ordered Materials School of Chemistry and Materials Science Gannan Normal University Ganzhou People's Republic of China; ^2^ Ordered Matter Science Research Center Nanchang University Nanchang People's Republic of China; ^3^ College of Chemistry and Materials Engineering Bohai University Jinzhou People's Republic of China

**Keywords:** flexible, hybrid perovskite, molecular ferroelectric, piezoelectricity, sensing

## Abstract

Piezoelectric materials for wearable biomechanical sensing require high flexibility, including the mechanical capacity to endure stretching and bending deformations, etc. However, existing strategies for flexible composites embedding typically brittle piezoelectric crystals into polymer matrices still suffer from limited stretchability and mechanical strength, falling short of dynamic, all‐day wearable scenarios. Herein, we synthesize a new luminescent organic‐inorganic hybrid perovskite ferroelectric molecular crystal, *N,N*‐dimethylallylammonium MnCl_3_ (DMAA‐MnCl_3_), which exhibits a transverse piezoelectric response of about 36 pC/N and a higher Curie temperature (*T*_c_ = 357 K) than that of its Cd analog (Δ*T* = +18 K; Wang et al. *J. Am. Chem. Soc*. 2020, 142, 12857). By in situ growing DMAA‐MnCl_3_ microcrystals within a styrene‐ethylene‐butylene‐styrene/styrene‐isoprene‐styrene (SEBS/SIS) double‐polymer matrix, we fabricated a flexible ferroelectric composite SEBS/SIS/DMAA‐MnCl_3_ that delivers excellent mechanical properties (tensile strength up to 7.54 MPa, tensile strain > 1200%), strong red emission, and reliable piezoelectric sensing for motion monitoring over 10 000 cycles. Notably, it maintains stable sensing responses under harsh conditions such as high humidity (100% RH) and subzero temperatures (as low as −35°C), opening prospects for all‐weather environmental scenes. This work provides a practical design strategy for flexible ferroelectric composites with promising application prospects.

## Graphical abstract

A flexible molecular ferroelectric composite is fabricated via in situ growth of luminescent DMAA‐MnCl_3_ ferroelectric crystals within a SEBS/SIS double‐polymer matrix. It delivers 7.54 MPa tensile strength, over 1200% tensile strain, durable piezoelectric motion sensing, and excellent harsh‐environment stability for all‐weather wearable biomechanical monitoring.

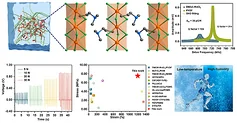

## 1 Introduction

Piezoelectric materials, capable of exhibiting electric potentials in response to mechanical stress due to their non‐centrosymmetric structures, have spurred growing interest across diverse applications spanning wireless communication, tissue engineering to soft robotics [1, 2, 3]. Conventional inorganic perovskite ferroelectrics, including lead zirconate titanate (PZT) and barium titanate (BTO), have long served as the cornerstone of sensor, transducer, and energy harvester technologies, attributed to their superior piezoelectric coefficients (e.g., high *d*_33_ values) and robust stability [4, 5, 6, 7]. In parallel, ferroelectric molecular crystals have emerged as a compelling family, offering distinct merits such as structural designability, solution processability, biocompatibility, and environmental friendliness [8, 9]. These advantages have expanded their functional scope from conventional sensing and memory devices to cutting‐edge interdisciplinary investigations like wearable biomechanical sensing and implantable bioelectronics [10, 11, 12]. Notable examples of high‐performance ferroelectric molecular crystals include TMCM‐CdCl_3_ (TMCM = trimethylchloromethyl ammonium, *d*_33_ = 383 pC/N) [13], NDABCO‐NH_4_‐Br_3_ (NDABCO = *N*‐amino‐*N*′‐diazabicyclo [2.2.2]‐octonium, *d*_33_ = 63 pC/N) [14], and HFPD (HFPD = perfluoro‐1,5‐pentanediol, *d*_33_ = 138 pC/N) [15] etc., Crucially, they intrinsically exhibit excellent piezoelectricity without pre‐stretching or poling required for piezoelectric polymers and ceramics [16, 17, 18]. Nevertheless, such crystalline materials are typically brittle with limited elastic recovery, depressing their direct use in emerging scenarios such as flexible electronics and wearable devices.

To address these limitations, embedding ferroelectric molecular crystals into flexible polymer matrices retains the intrinsic piezoelectric response of the crystals while imparting deformability and mechanical robustness to the material through the polymer matrix [19, 20, 21, 22]. Nevertheless, current research largely prioritizes single‐property optimization, falling short in the coordinated balance of multiple functions. For example, composites such as γ‐Glycine/PVA (PVA = polyvinyl alcohol) [23] and HFPD/PVA [15] merely attain deformability while preserving piezoelectric performance; Ren et al. further developed an IM‐in‐PVA (IM = imidazole perchlorate) ferroelectric hydrogel incorporating self‐healing functionality, which maintains stable ferroelectric performance under 20% strain [24]. The flexible ferroelectric composites TMCM‐CdCl_3_ [25] and CMDABCO‐NH_4_‐[ClO_4_]_3_ (CMDABCO = *N*‐chloromethyl‐*N*′‐diazabicyclo[2.2.2]octonium) [26] in a polydimethylsiloxane (PDMS) matrix show excellent piezoelectric sensing. However, their mechanical strength and stretchability remain at a low level, which is inadequate for high‐motion scenarios considering frequent deformations and physical damage. In the pursuit of ultra‐high stretchability, the HFPD/WPU (WPU = waterborne polyurethane) piezoelectric elastomer designed by Hu et al. achieves stable output at 200% strain, yet exhibits a limited tensile strength of merely 0.6 MPa, which severely restricts its mechanical load‐bearing capacity [27]. Most notably, Wu et al. incorporated moisture‐sensitive TMCM‐MnCl_3_ into an SEBS matrix, yielding a flexible composite that retains both piezoelectricity and photoluminescence while achieving a tensile strain exceeding 1300% with fracture strength up to 4 MPa [28]. Nevertheless, such piezoelectric composite systems still face potential challenges, particularly under conditions that may induce a toughness‐brittleness transition, such as degradation of mechanical properties and sensing deterioration under subzero temperature (< –30°C) or high‐humidity (> 90% RH) [29, 30]. These concerns highlight the ongoing vision of developing flexible piezoelectric composites capable of maintaining robust mechanical integrity alongside reliable functionality in harsh environments.

In this work, we reported a new perovskite ferroelectric molecular crystal, DMAA‐MnCl_3_, by a chemical substitution strategy replacing toxic B‐site Cd metal in DMAA‐CdCl_3_ with environmentally benign Mn under the guidance of ferroelectrochemistry. The obtained crystal exhibits enhanced red color emission and an 18 K increase in Curie temperature (*T*_c_ = 357 K), significantly broadening its operative window. By employing in situ growth of DMAA‐MnCl_3_ within a SEBS/SIS double‐polymer matrix through multiple molecular interactions, we fabricated a flexible ferroelectric composite SEBS/SIS/DMAA‐MnCl_3_. This composite demonstrates high tensile strength (7.54 MPa) and stretchability (> 1200%) due to strong SEBS/SIS chain entanglement, while the dense polymer network ensures stable and reliable piezoelectric motion sensing under harsh conditions (as low as –35°C and 100% RH) over 10 000 cycles. This work offers a viable design strategy for flexible piezoelectric sensors based on ferroelectric molecular crystals, showcasing potential in demanding health‐monitoring applications under extreme environments.

## 2 Results and Discussion

### 2.1 Crystal Structure and Ferroelectric Properties

A large number of orange DMAA‐MnCl_3_ crystals were obtained via slow evaporation of the mixed acetonitrile/methanol (3:1) solution of DMAA chloride and MnCl_2_·4H_2_O at 60°C. At 290 K (low‐temperature phase, LTP), DMAA‐MnCl_3_ adopts an orthorhombic noncentrosymmetric space group *Pna*2_1_ (no. 33, point group *mm*2) (Table S1). The crystal structure is composed of organic DMAA cations and anionic chain‐like [MnCl_3_]_n_^−^ frameworks aligned along the *c*‐axis (Figure 1a,b). The adjacent MnCl_6_ octahedra share a face, and the organic DMAA cations are located between the channels of the inorganic [MnCl_3_]_n_^−^ chains via hydrogen bonds N─H···Cl interactions (bond length of 2.51 Å) to present a 1D hybrid perovskite structure (Table S2), forming a similar stacking manner to that of hexagonal perovskite BaNiO_3_ [31]. The lengths of the Mn─Cl bond (2.5374(12)−2.5807(12) Å) and the adjacent Cl─Mn─Cl angles in a range from 82.29(4) to 97.87(4)° are comparable to those of other manganese‐chloride ABX_3_ hybrid perovskites, but they are shorter than those in the analog DMAA‐CdCl_3_, showing a large torsion of the octahedron and a small distortion parameter (Σ = 76.03°) (Figure S1 and Table S3) [32]. Additionally, the organic DMAA cations occupy a general position with no specific symmetry operations in the crystal structure and thus present an orderly state (Figure 1a). Upon heating to 383 K (high‐temperature phase, HTP), DMAAMnCl_3_ retains its orthorhombic crystal system but transitions to a higher centrosymmetric space group, *Pnma* (no. 62, point group *mmm*) (Table S1). The changes in the inorganic skeleton between this HTP and the LTP are minimal (Figure 1c). Notably, the shift in the crystal space group introduces the formation of new mirror symmetry within the crystal structure. In the HTP, the DMAA cation aligns precisely with this mirror plane, displaying orientational disorder where all its constituent atoms are uniformly distributed across the plane that intersects through the central N atom (Figure 1c,d). This structural evolution, marked by a symmetry transformation from *Pnma* to *Pna*2_1_, indicates a paraelectric‐to‐ferroelectric phase transition characterized by the Aizu notation *mmm*F*mm*2.

**FIGURE 1:**
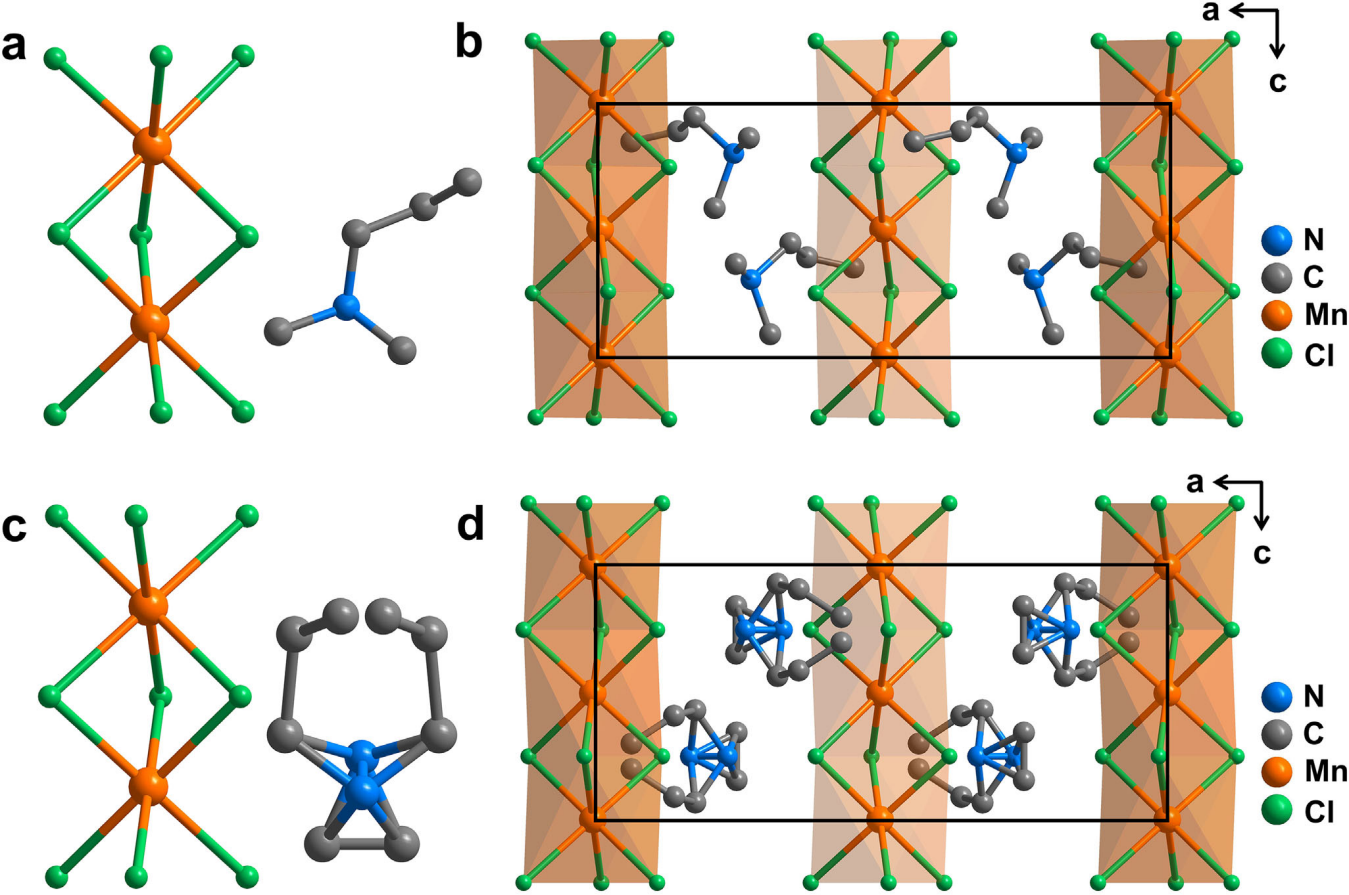
Molecular structure and packing view of DMAA‐MnCl_3_ plotted along the *b*‐axis in the LTP (a,b) and in the HTP (c,d). The hydrogen atoms of the DMAA cations were omitted for clarity.

DMAA‐MnCl_3_ also exhibits notable photoluminescent (PL) performance rooted in the octahedral coordination of Mn^2+^ ions and emits intense red light (∼650 nm) upon ultraviolet excitation with a photoluminescence quantum yield (PLQY) of 68.77% and a decay lifetime of 816 µs at room temperature (Figure S2) [33]. Its excitation and emission spectra further reveal three distinct excitation bands at 375, 450, and 540 nm, paired with a prominent emission peak at ∼650 nm. The crystal exhibits unchanged luminescent features under varied polarization and mechanical deformation (Figure S3). These spectral features are assigned to Mn^2+^ electronic transitions: ^6^A_1g_→^4^T_2g_(D), ^6^A_1g_→^4^T_2g_(G), and ^6^A_1g_→^4^T_1g_(G), respectively (Figure S4) [34]. Notably, the emission wavelength remains unchanged across excitation wavelengths spanning 345–480 nm, confirming that the PL emission is decoupled from the incident light wavelength and governed exclusively by Mn^2+^‐centered electronic transitions.

The phase transition characteristics of DMAA‐MnCl_3_ were further validated using differential scanning calorimetry (DSC) measurements. As depicted in Figure 2a, DMAA‐MnCl_3_ exhibits a pair of thermal anomalies at 357 and 349 K upon heating and cooling, far below the decomposition temperature of 470 K (Figure S5), confirming a structural phase transition at *T*_c_ of 357 K. The *T*_c_ is significantly higher than that of DMAA‐CdCl_3_ (*T*_c_ = 339 K, Δ*T* = +18 K). As illustrated in Figure S6, the contracted unit cell of DMAA‐MnCl_3_ indicates a more tightly packed molecular packing, which partly restricts the dynamic freedom of organic components, which in turn elevates the energy barrier required for phase transition, thereby correlating with its elevated transition temperature [35, 36]. The temperature‐dependent real part of dielectric permittivity (ε′) is highly sensitive to detecting structural phase transitions, in which a hallmark of such transitions is a dramatic variation in the dielectric constant near *T*_c_ [37]. In Figure 2b, DMAA‐MnCl_3_ reveals pronounced anomaly peaks with a twofold change in ε′ value at around 358 K during heating. Upon cooling, the ε′ exhibits a peak shape anomaly around 346 K, similar to that in a heating run. The reversible dielectric transition matches well with the DSC results. This highlights that the ε′ shows sharp *λ*‐shaped peaks approximately eightfold changes in value at 0.5 kHz, which is consistent with the characteristic ferroelectric phase transition (Figure 2c). Furthermore, the dielectric response adheres to the Curie−Weiss relationship in a paraelectric‐ferroelectric phase transition: ε′ = *C*_para_/(*T* – *T*_0_) (for *T* > *T*_c_, paraelectric phase) and ε′ = *C*_ferro_/(*T*_0_′ − *T*) (for *T* < *T*_c_, ferroelectric phase), where *C*_para_ and *C*_ferro_ are Curie constants, and *T*_0_/*T*_0_′ are Curie−Weiss temperatures for the respective phases [38]. Fitting these expressions yields *C*_para_ = 337 and *C*_ferro_ = 181, resulting in a *C*_para_ / *C*_ferro_ ratio of ∼1.86 (Figure 2c, inset), which tends to be a second‐order ferroelectric transition around 358 K. Correspondingly, the fitted Curie−Weiss temperatures are *T*_0_ = 356.44 K and *T*_0_′ = 356.62 K.

**FIGURE 2:**
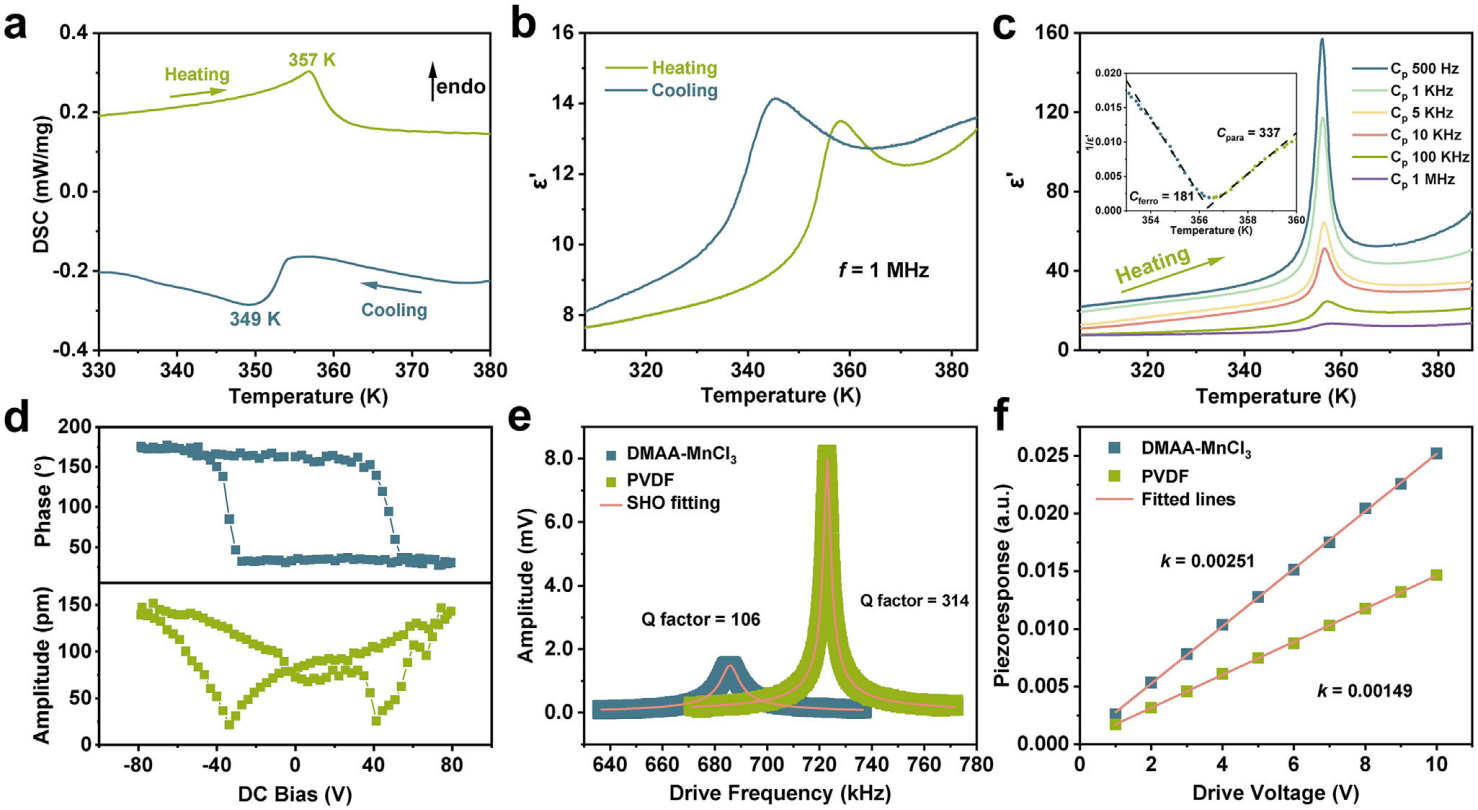
(a) DSC curves for DMAA‐MnCl_3_ in a heating‐cooling run. (b) Dielectric constant ε′ of DMAA‐MnCl_3_ at 1 MHz in a heating‐cooling run. (c) Variation of the dielectric constant ε′ of DMAA‐MnCl_3_ as a function of temperature during the heating process. (d) PFM phase and amplitude hysteresis loops. (e) Lateral piezoresponse as a function of frequency for the DMAA‐MnCl_3_ crystal and PVDF film, and analyzed by the SHO model. (f) Piezoelectric amplitude of the DMAA‐MnCl_3_ crystal and PVDF was plotted against the drive voltages.

To further validate the ferroelectric properties of DMAA‐MnCl_3_, we performed piezoresponse force microscopy (PFM) measurements. Lateral PFM phase (Figure S7a) and amplitude (Figure S7b) images reveal a well‐defined irregular domain structure in the phase channel, with domain walls appearing as dark contrast in the amplitude image. Crucially, the observed domain pattern is independent of the crystal's surface topography (Figure S7c), ruling out artifacts from morphological features. In Figure 2d, switching spectroscopy PFM curves exhibit a typical square‐shaped phase hysteresis loop and a butterfly‐shaped amplitude hysteresis loop [39 ^–^ 41]. These characteristics indicate that the polarization of DMAA‐MnCl_3_ is reversible under an applied electric field. The existence of domain structures and the reversibility of polarization collectively provide robust evidence confirming the ferroelectric nature of DMAA‐MnCl_3_. We further employed PFM to assess the piezoelectric response of the DMAA‐MnCl_3_ crystal. An AC tip bias was applied to excite piezoelectric vibrations near the resonant frequency. These measurements were carried out in lateral PFM mode, with the direction of the crystal oriented perpendicular to the PFM cantilever, thereby capturing the piezoelectric component *d*_31_. Figure 2e compares the lateral PFM amplitude as a function of driving frequency for both DMAA‐MnCl_3_ and a PVDF reference (*d*_31_ = 21.4 pC/N, measured at 10 V AC bias) [42]. Both exhibit sharp resonance peaks, well‐fit by the simple harmonic oscillator (SHO) model:

(1) $$\frac{A\left(\omega \right)=A_{\max}\omega_{0}^{2}/Q}{\sqrt{{\left(\omega_{0}^{2}-\omega^{2}\right)}^{2}+{\left(\omega_{0}^{2}\omega /Q\right)}^{2}}}$$

where *A* represents the amplitude, *ω*/*ω*_0_ denotes the driving and resonance frequency, respectively, *A*_max_ is the amplitude at the resonance frequency, and *Q* is the quality factor. The effective amplitude at resonance can be calculated as *A*_max_/*Q*. To determine the piezoelectric coefficient *d*_31_ of DMAA‐MnCl_3_, the *Q* factor‐corrected piezoelectric amplitudes of both materials were plotted against the applied drive voltages. As shown in Figure 2f, both plots exhibit a linear relationship between amplitude and voltage, confirming an intrinsic piezoelectric response. The slope of each line reflects the relative magnitude of the piezoelectric coefficient. Based on the relative slopes, we estimate the *d*_31_ coefficient of DMAA‐MnCl_3_ to be 36.05 pC N^−1^. To further clarify the intrinsic piezoelectric properties, we performed density functional theory (DFT) calculations to predict the piezoelectric coefficients of the crystals (Figure S8). These calculations yielded a small longitudinal piezoelectric *d*_33_ value of 4 pC N^−1^, closely matching the experimental value of 2.8 pC N^−1^ obtained via the ‘Berlincourt’ method (Figure S9). Additionally, the calculated piezoelectric *d*_31_ value was 27 pC N^−1^, slightly smaller than the in‐plane piezoelectric response measured by PFM.

### 2.2 Fabrication of SEBS/SIS/DMAA‐MnCl_3_ Composite

Bulk DMAA‐MnCl_3_ crystals show intrinsic brittleness, which severely limits their applications in wearable piezoelectric sensing for health monitoring. To obtain a flexible ferroelectric composite with superior mechanical properties, we fabricated a SEBS/SIS dual‐network matrix with DMAA‐MnCl_3_ as the piezoelectric active filler. First, we systematically investigated the synthesis of the SEBS/SIS dual‐network polymer matrix. A 10:9 mass ratio of SEBS to SIS yielded optimal mechanical properties in SEBS/SIS that exhibited a tensile strength of 14.57 MPa and could sustain a 7.5 Kg load without damage, significantly higher than the pristine SEBS (9.01 MPa) and SIS (4.31 MPa) monomers, respectively (Figures S10 and S11). When the two types of polymer networks are mixed, SIS chains are capable of interacting with SEBS chains via intermolecular forces, thus inducing extensive chain entanglement via a physical cross‐linking manner to fabricate remarkably enhanced mechanical properties. Physical crosslinking‐induced chain entanglement enhances 3D network compactness and reduces swelling performance in organic molecular chain systems. Swelling tests of SEBS/SIS blends and neat individual components (Figure S12) confirm chain entanglement, with a minimum SR of 65.57% achieved at a 10:9 SEBS/SIS mass ratio. Notably, SEBS/SIS retains excellent flexibility and deformability, enabling full recovery of the original shape after 700% tensile strain (Figure S13). Furthermore, the SEBS/SIS demonstrates remarkable compression resistance and excellent recovery capacity following compression. As shown in Figure S14, it maintained a high compressive strength of 87.26 MPa while exhibiting minimal energy dissipation (86.75 MJ m^−3^) and a low dissipation ratio of 3.2% at 80% strain. Meanwhile, the tensile repair tests of SEBS/SIS demonstrated excellent fatigue resistance during 1000 cycles of loading (Figure S15).

Subsequently, we fabricated the SEBS/SIS/DMAA‐MnCl_3_ flexible ferroelectric composite by in situ growth of DMAA‐MnCl_3_ crystals within the SEBS/SIS polymer matrix. In Figures S16a, SEM images and EDS mapping of the SEBS/SIS/DMAA‐MnCl_3_ composite reveal a uniform distribution of the ferroelectric filler. The spatial confinement effect of the 3D polymer network suppresses crystal growth, reduces grain size, and yields a homogeneous microcrystal arrangement (Figure S16b). Simultaneously, the polymer network does not affect the crystal structure and photoluminescence, and only serves as a flexible substrate (Figure S17). XRD analysis further confirms the crystalline state of these crystals embedded in the composite (Figure S18), in which multiple intermolecular interactions form between the structures of DMAA‐MnCl_3_ and the SEBS/SIS: specifically, Cl atoms in [MnCl_3_]^−^ engage in monopole–monopole interactions with ─CH_3_ groups on the polymer chains, while abundant van der Waals forces exist between [DMAA]^+^ and the SEBS/SIS polymer backbones (Figure 3a). Molecular dynamics (MD) simulation was carried out to elucidate the inherent microscopic interactions within the as‐fabricated composite system, revealing the interaction distances of C─H···Cl as 2.3593 and 2.6893 Å (Figure S19), both of which are shorter than the characteristic length scale of canonical noncovalent interactions (2.70–3.30 Å), enabling the formation of monopole–monopole interactions at the composite interface. Besides, abundant van der Waals interactions exist between [DMAA]^+^, SEBS/SIS chains, and [MnCl_3_]^–^. To directly validate the aforementioned microscopic interactions, Fourier transform infrared (FT‐IR) spectroscopy and X‐ray photoelectron spectroscopy (XPS) were further performed. The asymmetric bending vibration peak of the C─H bond associated with the ─CH_3_ moieties in the polymer matrix exhibits a distinct red shift from 1400 to 1389 cm^−1^ in the SEBS/SIS/DMAA‐MnCl_3_ composite (Figure S20a). Moreover, the dominant Cl 2p peak of the pristine DMAA‐MnCl_3_ crystal is centered at a binding energy of 198.45 eV, which undergoes a pronounced negative binding energy shift to 197.71 eV, corresponding to a total shift magnitude of 0.74 eV upon compounding with SEBS/SIS (Figure S20b). This reduction in binding energy is indicative of an elevated electron cloud density around the Cl atoms, providing further corroboration of the existence of monopole–monopole interactions. The numerous interactions between the filler and the matrix enhance interfacial adhesion, thereby enabling the ferroelectric composite to maintain a tensile strength of 7.54 MPa (Figure 3b). This value is derived from the 5 wt.% SEBS/SIS/DMAA‐MnCl_3_ ferroelectric composite, which exhibits the best piezoelectric performance and is detailed below. Moreover, it still retains outstanding tensile/compressive recoverability comparable to the pristine SEBS/SIS matrix (Figure 3c and Figure S21). In comparison with conventional flexible ferroelectric composites, the SEBS/SIS/DMAA‐MnCl_3_ flexible ferroelectric composite exhibits the maximum tensile strength, while its deformability also ranks among the leading levels; this collectively demonstrates that the composite material possesses exceptionally high toughness (Figure 3d) [15, 25, 27, 28, 43, 44, 45, 46, 47, 48, 49, 50, 51]. Under UV light irradiation, SEBS/SIS/DMAA‐MnCl_3_ flexible composite in both initial and stretched states exhibits bright red‐light emission (Figure S22), further confirming the successful fabrication of the ferroelectric composite with the uniform dispersion of DMAA‐MnCl_3_ filler in the polymer matrix.

**FIGURE 3:**
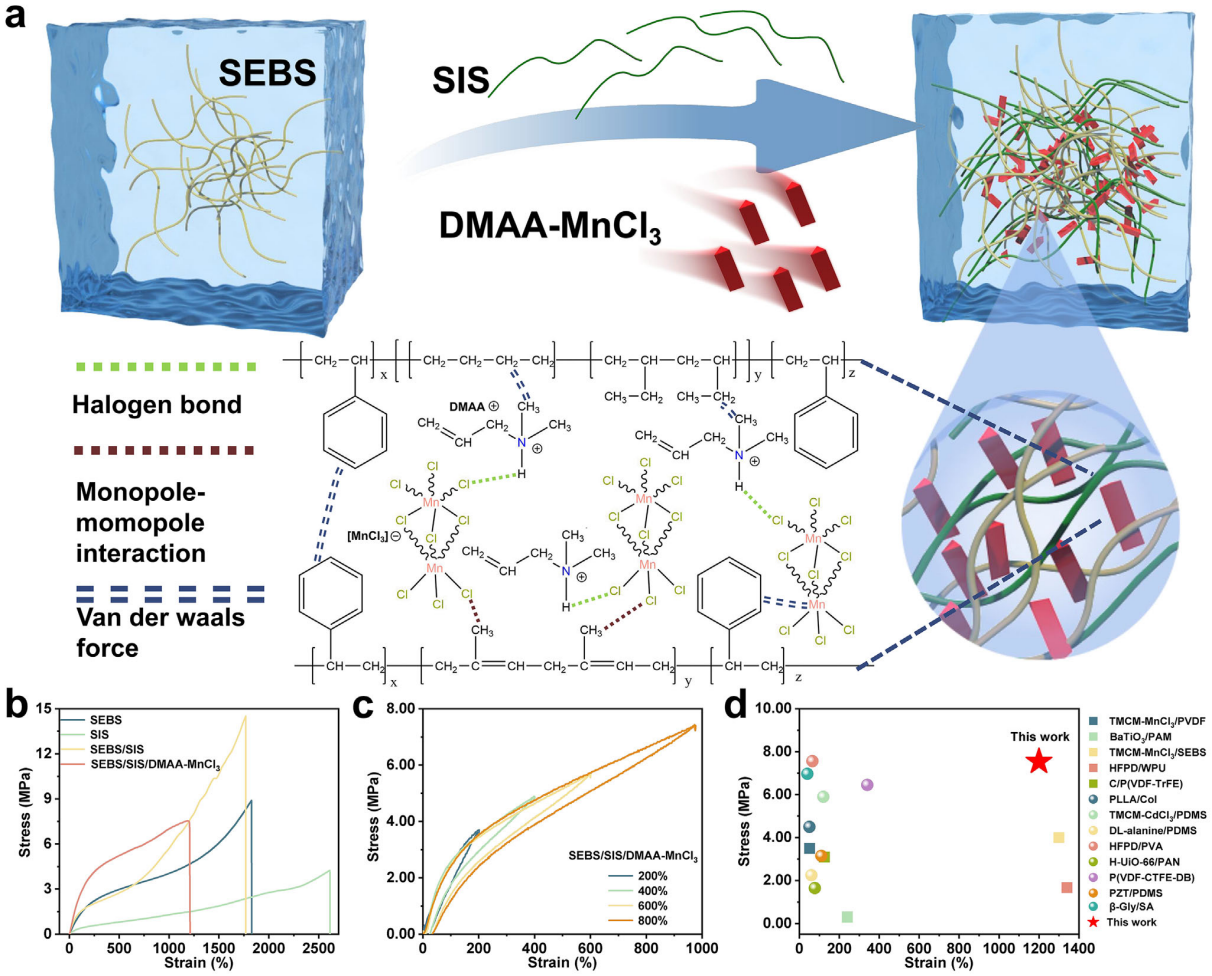
(a) Schematic diagram of the SEBS/SIS/DMAA‐MnCl_3_ composite. (b) Stress–strain curves of SEBS, SIS, SEBS/SIS, and SEBS/SIS/DMAA‐MnCl_3_ composite. (c) Loading‐unloading stress curves of the SEBS/SIS/DMAA‐MnCl_3_ composite. (d) Comparison of tensile stress and tensile strain in SEBS/SIS/DMAA‐MnCl_3_ composite and previously reported ferroelectric composite.

### 2.3 Piezoelectric Properties of SEBS/SIS/DMAA‐MnCl_3_ Composite

The composite's superior mechanical performance supports the practical application of its flexible sensors. To systematically evaluate the piezoelectric performance of the composite, we first fabricated a series of piezoelectric sensors based on flexible ferroelectric composites (Figure S23) with varying contents of SEBS/SIS polymer matrix and DMAA‐MnCl_3_ crystal fillers [52]. Then we tested their sensing response under 30 N pressure (Figure 4a and Figure S24). Specifically, the pure SEBS/SIS polymer matrix exhibits no piezoelectric effect, whereas a 5 wt.% DMAA‐MnCl_3_ crystal loading yielded the highest response of open‐circuit voltage (0.9546 V), indicating that the piezoelectricity stems solely from the DMAA‐MnCl_3_ crystals. It should be noted that the decrease in open‐circuit voltage when loading DMAA‐MnCl_3_ at a content exceeding 5 wt.% may be attributed to crystal aggregation, which compromises dispersion uniformity. Additionally, the 5 wt.% composite exhibited a higher short‐circuit current (137 nA) and optimal mechanical properties (Figure S25). This composition was therefore selected as the target for subsequent studies. Accordingly, this optimal content of DMAA‐MnCl_3_ was selected as the target for subsequent investigations. Additionally, when uniaxial compressive forces of 5, 10, 20, and 30 N are applied to this SEBS/SIS/DMAA‐MnCl_3_ composite, it produces a stable, linearly increasing open‐circuit voltage from 0.2 V to the peaking value at 0.9546 V (Figure 4b).

**FIGURE 4:**
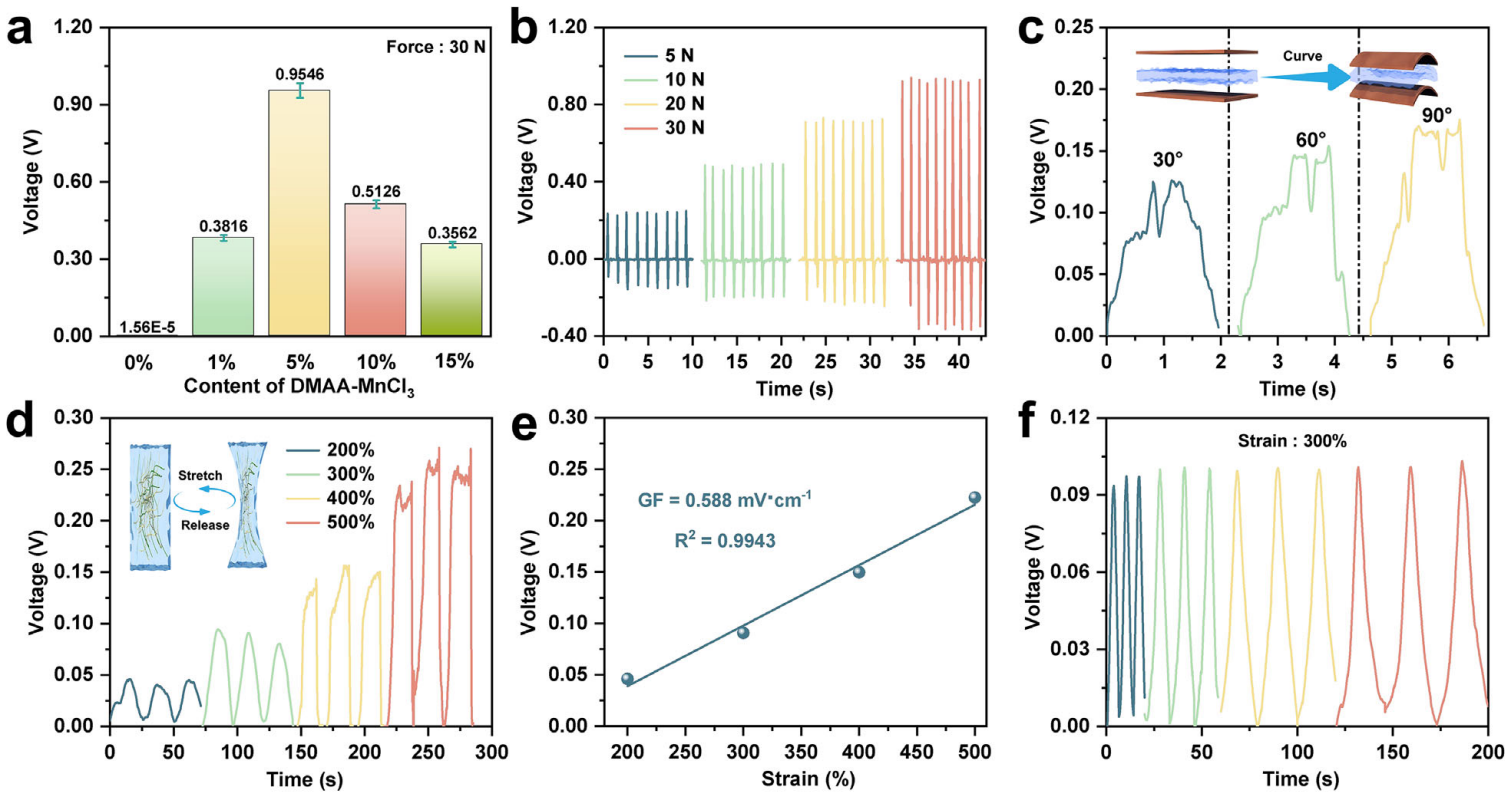
(a) Piezoelectric responses of flexible composites with different DMAA‐MnCl_3_ contents under 30 N pressure. (b) Voltage response curves of the SEBS/SIS/DMAA‐MnCl_3_ composite under different pressures (5, 10, 20, and 30 N). (c) Voltage response curves of the SEBS/SIS/DMAA‐MnCl_3_ composite at 30°, 60°, and 90° bending angles. (d,e) Voltage response curves and GF values of the SEBS/SIS/DMAA‐MnCl_3_ composite under 200%–500% strain. (f) Voltage response curves of the SEBS/SIS/DMAA‐MnCl_3_ composite at different stretching frequencies under 300% strain.

We further extended our evaluation to the piezoelectric responses of SEBS/SIS/DMAA‐MnCl_3_ composite under bending and tensile deformations. When bent from 30° to 90°, the flexible composite exhibited an increase in peak open‐circuit voltage from 0.1204 to 0.1658 V (Figure 4c). For tensile testing, the peak voltage of the SEBS/SIS/DMAA‐MnCl_3_ composite grew with increasing tensile strain, up to 0.2514 V at a 500% strain (Figure 4d). Strain yields a gauge factor (GF) of 0.588 mV cm^−1^ and an *R* ^2^ value of 0.9943, confirming excellent linearity (Figure 4e) [53]. The GF value of this composite exceeds that of other composites with different crystal loadings (e.g., 1 wt.% loading, GF = 0.4379 mV cm^−1^) (Figure S26). Even as tensile frequency increased gradually, the composite retained a stable piezoelectric output (Figure 4f). This frequency‐independent stability ensures reliable, continuous signal transmission in dynamic, variable motion environments. Additionally, the short‐circuit current of the SEBS/SIS/DMAA‐MnCl_3_ composite shows linear variations with changes in both compressive force and tensile strain. (Figure S27).

### 2.4 Piezoelectric Responses in Harsh Conditions

Complex environmental stability is critical for the long‐term reliability of functional devices [54, 55]. First, we evaluated the flexibility and luminescence of the SEBS/SIS/DMAA‐MnCl_3_ composite under both subzero (−35°C) and ambient (20°C) conditions (Figure 5a). The retained stretchability, twistability, and luminescence in the cold environment confirm no damage to either the polymer matrix or ferroelectric fillers from low‐temperature exposure. To further assess its piezoelectric response at low temperatures, we characterized the piezoelectric voltage output under 300% strain at −35°C (Figure 5b), with a tiny reduction in peak voltage. As the temperature dropped to −40°C, the open‐circuit voltage of the composite exhibited a non‐negligible decrease (Figure S28). We then tracked its piezoelectric output over 3 days of continuous low‐temperature operation (Figure 5c). The signal remained undistorted and consistent with the initial value. Under frozen conditions, gradient tensile tests exhibit linear fitting with the GF value remaining at 0.537 mV cm^−1^, comparable to that in ambient conditions (Figure 5d,e).

**FIGURE 5:**
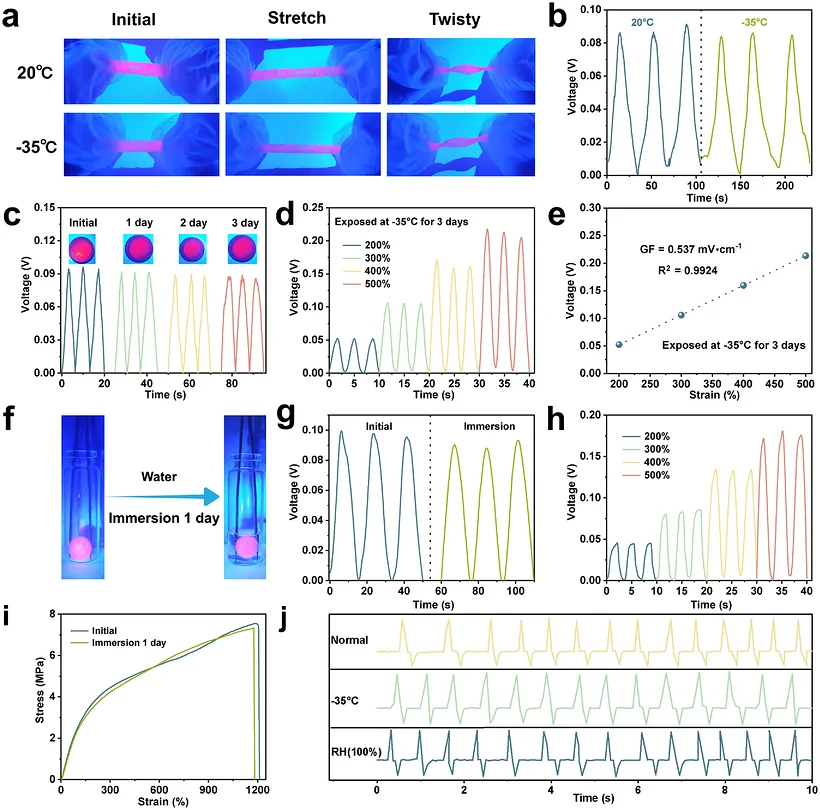
(a) Comparative photographs of SEBS/SIS/DMAA‐MnCl_3_ composite showing stretching and twisting under room temperature and −35°C. (b,c) Voltage response curves of the SEBS/SIS/DMAA‐MnCl_3_ composite under varying temperatures and after 3‐day storage at −35°C. (d,e) Voltage response curves and GF values of the SEBS/SIS/DMAA‐MnCl_3_ composite tested at −35 °C under 200%–500% tensile strain. (f,g) Comparative diagrams of luminescence behavior and voltage response curves of the SEBS/SIS/DMAA‐MnCl_3_ composite after 1‐day water immersion. (h,i) Voltage response curves and stress‐strain curves of the SEBS/SIS/DMAA‐MnCl_3_ composite under 200%–500% tensile strain following 1‐day water immersion. (j) Voltage response curve of the SEBS/SIS/DMAA‐MnCl_3_ composite under 30 N compressive load under low‐temperature and humid conditions.

Second, we subjected them to a high‐humidity environment for stability tests. Direct immersion of the SEBS/SIS/DMAA‐MnCl_3_ composite in water for 24 h (Figure 5f) resulted in no notable change in luminescence intensity upon UV light irradiation, indicating the ferroelectric fillers in the polymer matrix are still active. It should be noted that DMAA‐MnCl_3_ is a highly moisture‐sensitive material, indirectly indicating that the SEBS/SIS dual‐network polymer matrix can effectively prevent humidity from damaging the active ingredients. We then evaluated piezoelectric response post‐immersion (Figure 5g). The peak voltage did not change significantly compared to that at the pristine state. The piezoelectric responses show a linear increasing trend from 0.05 to 0.2 V corresponding to the tensile strain rising from 200% to 500% (Figure 5h). The calculated GF value is 0.478 mV cm^−1^ (Figure S29). Notably, mechanical tests still maintain robust performance with minimal reduction when subjected to a high‐humidity environment (Figure 5i). As shown in Figure 5j, piezoelectric responses under compressive loads in complex environments were also validated for stability.

### 2.5 Real‐Time Human Motion Sensing

This composite's outstanding mechanical, piezoelectric, and environmental performance indicates application potential and operational reliability demands further experimental verification. First, the in vitro cytotoxicity of the composite was assessed to evaluate the potential biocompatibility. We employed the murine preosteoblast MC3T3‐E1 cell line and the fibroblast L929 cell line. Confocal fluorescence microscopy revealed only a negligible population of dead cells (labeled by red fluorescence), with the cellular viability of both cell lines remaining above 95% (Figure S30). Then, to advance the practical deployment of the SEBS/SIS/DMAA‐MnCl_3_ composite in human motion sensing, we prioritized evaluating its long‐term cyclic stability, a critical challenge limiting the durability of flexible sensors during prolonged use (Figure 6a) [56]. Impressively, it maintained excellent structural integrity throughout the test, with the output voltage remaining remarkably stable (peak voltage degradation < 10%), even after being placed for 1 and 2 months at ambient conditions (Figure S31). Notably, the composite also exhibited superior stability in its mechanical properties, current signal, and luminescent behavior (Figure S32). After undergoing cyclic compression and bending tests, the composite also retained a stable output voltage (Figure S33), thereby meeting the demands of diverse motion modes in practical applications. The as‐prepared composite exhibits a signal‐to‐noise ratio (SNR) of 35.91 dB, as well as a response time (176.3 ms) and a recovery time (145.6 ms), reflecting its reliable signal recognition and anti‐interference capability (Figure S34). Leveraging these properties, we integrated the composite into a wearable sensor for real‐time multi‐joint human motion monitoring.

**FIGURE 6:**
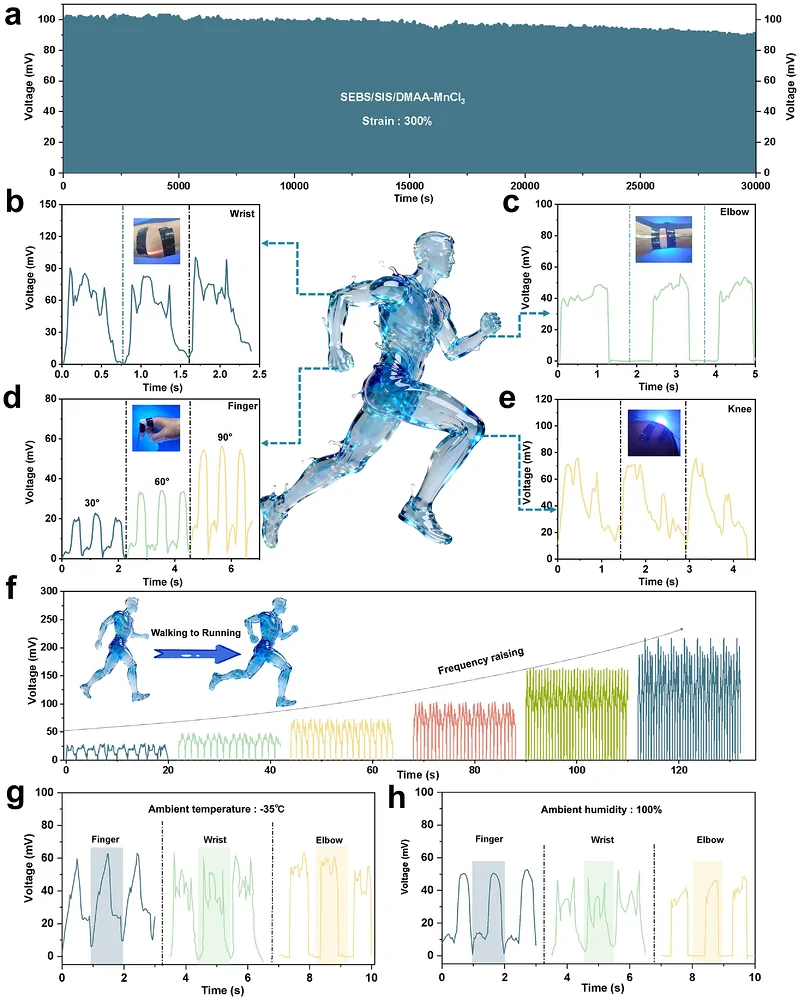
(a) Piezoelectric voltage outputs of the SEBS/SIS/DMAA‐MnCl_3_ composite during 10 000 consecutive cyclic loading‐unloading tests. (b–f) Real‐time monitoring of movements in finger, elbow, wrist, and knee joints, alongside synchronous tracking of plantar gait frequency, using the SEBS/SIS/DMAA‐MnCl_3_ composite. (g,h) Real‐time monitoring of movements under low‐temperature and humid conditions.

Besides, we verified that the different sensor‐skin interfacial methods introduced no interference with the acquired signals (Figure S35). As shown in Figure 6b–e, the sensor successfully captured fine‐grained kinematic signals from multi‐joint human movements, including finger, elbow, wrist, and knee joints. Notably, when attached to the finger joint, the output voltage of the sensor showed a strong linear correlation with the bending angles, enabling precise quantification of subtle hand movements (Figure 6d). It also demonstrated exceptional dynamic responsiveness, enabling clear differentiation between fast and slow motions (Figure 6f). Furthermore, continuous motion detection tests were conducted with a 0.4% NaCl artificial sweat solution applied to simulate the impacts of skin perspiration, prolonged vigorous exercise, and wear‐induced friction on sensing performance. Real‐time voltage recording during the test revealed that the sensor exhibited stable signal output under these simulated conditions (Figure S36). Beyond basic motion tracking, leveraging its freeze and water resistance, the flexible sensor maintained stability, showcasing its potential for all‐weather wearable health monitoring and outdoor activity tracking. These results collectively underscore the SEBS/SIS/DMAA‐MnCl_3_ composite as a promising candidate for next‐generation wearable biosensors (Figure 6g,h).

Systematic screening identified a matrix formulation (SEBS: SIS = 10:9 mass ratio) and filler content (5 wt.%) that together achieve simultaneous optimization of mechanical tensile strength (7.54 MPa), strain (> 1200%), and piezoelectric sensing performance, demonstrating the highest toughness among flexible ferroelectric composites reported to date (Figure 3d). The comprehensive sensing evaluation of SEBS/SIS/DMAA‐MnCl_3_ ferroelectric composite revealed its superior resistance to large‐amplitude, high‐frequency deformations, even under harsh conditions (e.g., high humidity and low temperatures), despite the moderate piezoelectric coefficient of the ferroelectric crystal DMAA‐MnCl_3_. Furthermore, the SEBS/SIS/DMAA‐MnCl_3_ composite exhibits exceptional cycling stability, maintaining stable sensing output over 10 000 cycles. This outperforms most previously reported ferroelectric composites, such as HFPD/WPU [27], TMCM‐MnCl_3_/SEBS [28], H‐UiO‐66/PAN [48], and β‐Gly/SA [51], highlighting its potential as an alternative flexible ferroelectric material for all‐weather wearable piezoelectric sensing applications.

## 3 Conclusions

In summary, we report the successful synthesis of a red‐emitting perovskite ferroelectric molecular crystal, DMAA‐MnCl_3_, via the utilization of environmentally benign Mn^2+^ ions to replace toxic Cd^2+^ upon our prior work on ferroelectric DMAA‐CdCl_3_. This crystal demonstrates an in‐plane piezoelectric response (36 pC/N) as well as an 18 K elevation in *T*_c_ (357 K) and enhanced red photoluminescence, expanding its operational window beyond the original Cd‐based analogue. By leveraging in situ growth of DMAA‐MnCl_3_ microcrystals within a SEBS/SIS double‐polymer matrix, we engineer a flexible ferroelectric composite (SEBS/SIS/DMAA‐MnCl_3_) that transcends the brittleness and environmental instability of traditional molecular ferroelectrics. This composite delivers robust mechanical properties, including tensile strength (7.54 MPa) and tensile strain (> 1200%), while maintaining piezoelectric sensing stability at −35°C or under 100% RH. When integrated into human motion sensors, the composite demonstrates real‐time tracking capabilities for multi‐joint movements (fingers, elbows, and wrists) even in complex environments, validating its practical utility. This study demonstrates a paradigm for bridging molecular design with macroscopic functionality and paves the way for molecular ferroelectric materials to advance practical applications in all‐weather wearable health monitoring and beyond.

## 4 Experimental Section

### 4.1 Materials

*N,N*‐dimethylallylamine, Manganese (II) chloride tetrahydrate (MnCl_2_·4H_2_O), Hydrochloric acid (37%), Normal hexane, Methanol, Acetonitrile, and Styrene‐ethylene‐butylene‐styrene (SEBS) and Styrene‐isoprene‐styrene (SIS) were purchased from Shanghai McLean Biochemicals Ltd (China). All reagents and solvents were purchased and used without further purification.

### 4.2 Preparation of Ferroelectric Molecular Crystals

First, hydrochloric acid and *N,N*‐dimethylallylamine were weighed in a molar ratio of 1.2:1. Subsequently, hydrochloric acid was added dropwise to an ethanol solution of *N,N*‐dimethylallylamine at a rate of 1 drop/s under an ice‐bath condition. A large amount of white solid *N,N*‐dimethylallylammonium chloride (hygroscopicity) was obtained by evaporating the solvent by rotary evaporation at 60°C. Next, *N,N*‐dimethylallylammonium chloride and MnCl_2_·4H_2_O were dissolved in a methanol/acetonitrile mixed solvent (1:3 v/v) at a molar ratio of 1:1. The resulting solution was subjected to slow evaporation at 60°C for 24 h, finally yielding orange DMAA‐MnCl_3_ crystals.

### 4.3 Preparation of Ferroelectric Composite

First, SEBS and SIS were dissolved in 15 mL of normal hexane at different weight ratios. The resulting solution was then poured into a glass petri dish and allowed to stand under ambient conditions for 5 h to form the SEBS/SIS substrate. An aliquot of the methanol/acetonitrile solution obtained from the preparation of molecular crystals was cast onto the composite substrate. The system was subsequently maintained at 60°C to induce crystal precipitation on the gel surface. After complete precipitation, an equal volume of the SEBS/SIS normal hexane solution was reapplied over the surface, and the sample was left to stand under ambient conditions for another 5 h. Finally, three cycles of dissolution and drying were performed using normal hexane, yielding the SEBS/SIS/DMAA‐MnCl_3_ ferroelectric composite.

### 4.4 Measurements

Methods of single‐crystal X‐ray diffraction, TGA, XRD, DSC, PFM measurements, SEM, MD simulations, DFT calculations, cytotoxicity test, mechanical, and piezoelectric response measurements were described in the Supporting Information.

## Author Contributions

**Shuangqing Li**: writing – original draft, data curation. **Zhe‐Kun Xu**: data curation. **Zheng Xing**: data curation. **Jin‐Wang Liu**: data curation, writing – review and editing. **Yong‐Quan Wu**: data curation, writing – review and editing. **Peng‐Fei Li**: writing – original draft, writing – review and editing. **Zhong‐Xia Wang**: writing – review and editing, conceptualization, supervision, funding acquisition.

## Funding

This work was supported by the National Natural Science Foundation of China (22222502) and the Research Team Program of Gannan Normal University.

## Conflicts of Interest

The authors declare no conflicts of interest.

## Supporting information




**Supporting File**: advs76643‐sup‐0001‐SuppMat.docx.

## Data Availability

The data that support the findings of this study are available from the corresponding author upon reasonable request.
