# Supplementary material for: Stretchable, Tough, and Luminescent Perovskite Molecular Ferroelectric Composite: Empowering Reliable Self‐Powered Motion Sensing in High Humidity and Subzero Temperatures
Source: Adv Sci (Weinh). 2026 Jul 20;13(56):e76643. doi: 10.1002/advs.76643 (PMC13383687; doi:10.1002/advs.76643)
Supplement: Supplementary file 1 [file ADVS-13-e76643-s001.docx]

Supporting Information

Stretchable, Tough, and Luminescent Perovskite Molecular Ferroelectric Composite: Empowering Reliable Self-Powered Motion Sensing in High Humidity and Subzero Temperatures

*Shuangqing Li, Zhe-Kun Xu, Zheng Xing, Jin-Wang Liu, Yong-Quan Wu, Peng-Fei Li,* Zhong-Xia Wang**

Measurement Methods

**Single-crystal X-ray diffraction:** Variable-temperature X-ray single-crystal diffraction data were collected on a Rigaku Saturn 724^+^ diffractometer with *Mo–Kα* radiation (λ = 0.71073 Å). Data processing, including empirical absorption corrections, was performed using the CrystalClear software package (Rigaku, 2018). The structures were solved by direct methods and refined by the full-matrix method based on F^2^ by means of the SHELXLTL software package. Non-H atoms were refined anisotropically using all reflections with *I* > 2σ (I). All H atoms were generated geometrically and refined using a "riding" model with U_iso_ = 1.2U_eq_ (C and N). The asymmetric units and the packing views were drawn with DIAMOND (Brandenburg and Putz, 2005). Angles and distances between some atoms were calculated using DIAMOND, and other calculations were carried out using SHELXLTL.

**PFM measurements:** PFM visualization of the ferroelectric domain structures was carried out using a commercial atomic force microscope system (MFP-3D, Asylum Research) on a thin-film sample. Conductive Pt/Ir-coated silicon probes (EFM-50) were used for domain imaging and polarization switching studies. Resonant-enhanced PFM mode was used to enhance the signal, with a typical AC voltage frequency of about 360 kHz and an AC amplitude of 10 V for the out-of-plane signal, with a 720 kHz AC voltage frequency and 2 V AC amplitude for the in-plane signal.

**Molecular dynamics simulations:** The DMAA-MnCl_3_ (100) crystal plane was cleaved as the adsorption substrate, with a 20 Å vacuum layer along the c-axis to eliminate inter-unit cell interference under periodic boundary conditions (PBC). Representative chain segments of SEBS and SIS were separately constructed and pre-optimized in an expanded vacuum supercell. Four interface adsorption models were built via manual assembly and low-energy conformation search: (1) SEBS on DMAA-MnCl_3_ (100) surface; (2) SIS on DMAA-MnCl_3_ (100) surface; (3) SEBS-SIS blending interaction model; (4) ternary composite system with SEBS and SIS co-adsorbed on DMAA-MnCl_3_ (100) surface.

**Basic characterization:** The ferroelectric composite's microscopic morphology was observed and recorded using a Hitachi S4800 benchtop scanning electron microscope with an accelerating voltage of 10 kV. The functional groups of the composite were characterized by Fourier transform infrared spectroscopy (FT-IR, Nicolet IS50, 400-4000 cm^-1^). X-ray photoelectron spectroscopy (XPS) was performed on a Thermo Scientific K-Alpha system (monochromatic Al Kα source, *hν* = 1486.6 eV; base pressure < 5×10^-10^ mbar). Surface charging of the insulating composite was compensated via a low-energy electron flood gun. Survey spectra (0-1300 eV) were collected at 100 eV pass energy (1.0 eV step), and high-resolution core-level spectra at 30 eV pass energy (0.05 eV step). All binding energies were calibrated to the adventitious C1s peak (284.8 eV), with peak deconvolution carried out in Thermo Avantage software using Shirley-type background correction. The mechanical properties of these ferroelectric composites were tested using a DR-509ASQ universal testing machine at room temperature. The ferroelectric composites that were prepared were cut into dumbbell shapes, with a length of 50 mm, a width of 2 mm, and a thickness of 1.2 mm. They were then tested at a stretching speed of 100 mm/minute. When conducting the compression performance test, the compression mold provided with the instrument was used instead. The composite was prepared into a cylinder with a radius of 10 mm and a height of 3 mm, while the movement rate of the instrument remained unchanged.

**Thermal analyses:** Differential scanning calorimetry (DSC) was performed by heating and cooling the polycrystalline samples on a Perkin–Elmer Diamond. The temperature range was 254–468 K with a heating rate of 30 K/min under a nitrogen atmosphere in aluminum crucibles. For thermal stability, thermogravimetric analysis (TGA) of DMAA-MnCl_3_ was characterized on a TGA-Q50 at a heating rate of 10 K/min in nitrogen.

**Swelling test:** The swelling test was performed as follows: A precisely weighed polymer sample was immersed in 20 mL of ethyl acetate for 5 h. After immersion, the sample was retrieved, residual surface solvent was gently blotted off with filter paper, and the sample was reweighed accurately. The swelling ratio (SR) was calculated from the sample mass change before and after immersion, with the corresponding formula shown below:

$$SR=\frac{W_{i}-W_{t}}{W_{i}}\times100\%$$

where W_t_ (g) and W_i_ (g) represent the mass of the polymer before and after it absorbs the solvent, respectively.

**Piezoelectric sensing:** The piezoelectric response of the composite was measured using a bench-top multimeter (DMM6500). Copper sheets were fixed on both sides of the composite sample (8 cm × 0.5 cm) and connected to the universal testing machine. The two ends of the sample were connected to the bench-top multimeter with copper sheets via wires. The voltage change (ΔV) was directly measured. The figure of merit (GF) can be used as an indicator of the piezoelectric response sensitivity, and its mathematical expression is:

$$GF=\frac{\Delta V}{\varepsilon}$$

The symbol "ΔV" represents the relative change in the composite response voltage (V - V_0_), while "ε" indicates the strain of the composite. Signal-to-Noise Ratio (SNR), as a core performance index of sensors, whose value directly determines the sensor's effective signal recognition capability and anti-interference ability against background noise; the unit of this index is decibel (dB), and its calculation formula and symbol definitions are given as follows:

$$SNR=20\lg\frac{A_{\mathrm{signal}}}{A_{\mathrm{noise}}}$$

A_signal_ denotes the peak value of the effective signal, and A_noise_ denotes the root-mean-square amplitude of the background noise. For piezoelectric performance tests under compression, bending, and tensile conditions, the test environment’s temperature and relative humidity were controlled at 20°C and 40%, respectively, with a control error of ≤5%. Only in the low-temperature tests was the relative humidity maintained at 20% (excessively high humidity would cause malfunction of the testing equipment).

**Sensor performance:** The as-prepared composite was cut into 50 mm × 5 mm strips, assembled into motion sensors by affixing copper electrodes with conductive leads to both strip ends and encapsulating electrode regions with polyimide tape. For motion sensing tests, target skin sites were pre-cleaned with 75% medical ethanol and air-dried; the sensor was adhered to the skin with its sensitive surface facing the epidermis, pressed for 10-20 s, and edge-secured with medical breathable tape (sensing area uncovered). Formal tests were conducted after pre-tests confirmed no sensor displacement and synchronous deformation with the skin.

**DFT calculations:** First-principles calculations were performed via DFT in the VASP package, using the PBE-GGA exchange-correlation functional and DFT-D3 (BJ) van der Waals corrections. A plane-wave energy cutoff of 550 eV and a 2×2×4 Monkhorst-Pack k-point mesh were adopted for all calculations. The elastic stiffness tensor was obtained via the finite difference method, and the piezoelectric constants via DFPT. Young’s and shear moduli were derived from the elastic stiffness matrix, with the 293 K experimental crystal structure used as the ground state for all property calculations.

The elastic stiffness constant of point group *mm*2 is presented as a 6 × 6 matrix:

$$\left[ \begin{matrix} \text{c}_{\text{11}} & \text{c}_{\text{12}} & \text{c}_{\text{13}} & \text{0} & \text{0} & \text{0} \\ \text{c}_{\text{21}} & \text{c}_{\text{22}} & \text{c}_{\text{23}} & \text{0} & \text{0} & \text{0} \\ \text{c}_{\text{31}} & \text{c}_{\text{32}} & \text{c}_{\text{33}} & \text{0} & \text{0} & \text{0} \\ \text{0} & \text{0} & \text{0} & \text{c}_{\text{44}} & \text{0} & \text{0} \\ \text{0} & \text{0} & \text{0} & \text{0} & \text{c}_{\text{55}} & \text{0} \\ \text{0} & \text{0} & \text{0} & \text{0} & \text{0} & \text{c}_{\text{66}} \end{matrix} \right]$$

The piezoelectric strain constants of point group *mm*2 can be described by a third-rank tensor in the form of a 3 × 6 matrix:

$$\left[ \begin{matrix} \text{0} & \text{0} & \text{0} & \text{0} & \text{d}_{\text{15}} & \text{0} \\ \text{0} & \text{0} & \text{0} & \text{d}_{\text{24}} & \text{0} & \text{0} \\ \text{d}_{\text{31}} & \text{d}_{\text{32}} & \text{d}_{\text{33}} & \text{0} & \text{0} & \text{0} \end{matrix} \right]$$

To characterize the mechanical behavior of DMAA-MnCl_3_ in its room-temperature ferroelectric phase, the elastic stiffness matrix *c*_ij_ was calculated from its single-crystal diffraction structure. In matrix notation, the stiffness tensor *c*_ij_ for the ferroelectric phase is given below:

$$\left[ \begin{matrix} \text{7.3} & \text{4.5} & \text{5.7} & \text{0} & \text{0} & \text{0} \\ \text{4.5} & \text{7.7} & \text{3.4} & \text{0} & \text{0} & \text{0} \\ \text{5.7} & \text{3.4} & \text{26.6} & \text{0} & \text{0} & \text{0} \\ \text{0} & \text{0} & \text{0} & \text{1.8} & \text{0} & \text{0} \\ \text{0} & \text{0} & \text{0} & \text{0} & \text{4.9} & \text{0} \\ \text{0} & \text{0} & \text{0} & \text{0} & \text{0} & \text{4.3} \end{matrix} \right]$$

The DMAA-MnCl_3_ crystal (*mm*2 point group) has an elastic stiffness matrix symmetric about the main diagonal with 12 independent non-zero components, and its low stiffness constants confirm intrinsic mechanical softness. Directional Young’s modulus (Figure S8a) shows strong anisotropy, with the c-axis value far exceeding those along the other two principal axes, originating from the 1D [MnCl_3_]^-^ framework extending along the c-axis. The shear modulus spatial distribution presents a shell-like structure, with the six-maximum MIN surface nested within the MAX surface (Figures S8b and S8c). The piezoelectric matrix of ferroelectric-phase DMAA-MnCl_3_ was also calculated. To compare with commonly used piezoelectric strain constants (*d*_ij_), the piezoelectric stress coefficients *e*_ij_ were converted to *d*_ij_ using the relation *d*_ij_ = *e*_ij_ * *s*_ij_, where the elastic compliance matrix *s*_ij_ is the inverse of the elastic stiffness matrix *c*_ij_. From the *e*_ij_ and *c*_ij_ matrices, the piezoelectric strain constants *d*_ij_ were directly derived as follows:

$$\left[ \begin{matrix} \text{0} & \text{0} & \text{0} & \text{0} & \text{4.2} & \text{0} \\ \text{0} & \text{0} & \text{0} & \text{-21.6} & \text{0} & \text{0} \\ \text{27.1} & \text{-18.2} & \text{-4.0} & \text{0} & \text{0} & \text{0} \end{matrix} \right]$$

The piezoelectric matrix reveals that the DMAA-MnCl_3_ crystal possesses a substantial transverse piezoelectric coefficient, with *d*_31_ reaching 27.1 pC/N, in close agreement with the experimentally measured value. Additionally, the calculated *d*_33_ value is 4.0 pC/N, which represents a moderate level among molecular systems.

**Cytotoxicity test:** The murine preosteoblast cell line MC3T3-E1 and murine fibroblast cell line L929 were routinely maintained in complete medium supplemented with 10% (v/v) fetal bovine serum and 1% (v/v) penicillin-streptomycin, in a humidified incubator at 37 ℃ with 5% CO_2_. Cells in the logarithmic growth phase were digested with trypsin-EDTA, subcultured, and employed for subsequent experiments. The sterilized test samples were placed flat at the bottom of 96-well plates, followed by seeding of MC3T3-E1 and L929 cell suspensions into the corresponding wells, respectively. After 24 h of incubation, the original culture medium in each well was carefully aspirated and replaced with fresh complete medium, and 20 μL of 3-(4,5-dimethylthiazol-2-yl)-2,5-diphenyltetrazolium bromide (MTT) solution at a concentration of 5 mg/mL was added to each well for an additional 4 h of incubation in the dark. Subsequently, the supernatant was carefully removed without disturbing the formed formazan crystals at the well bottom; 150 μL of dimethyl sulfoxide (DMSO) was added to each well, and the plates were horizontally shaken at room temperature in the dark for 10 min to fully dissolve the crystals. The absorbance (OD value) of each well at 490 nm was measured with a microplate reader, and the relative cell viability was calculated according to the following formula:

$$\text{V}\text{=}\text{(}\text{OD}_{\text{e}}\text{-}\text{OD}_{\text{o}}\text{)/(}\text{OD}_{\text{c}}\text{-}\text{OD}_{\text{o}}\text{)}$$

where OD_e_ represents the value of the experimental group, OD_o_ denotes the value of the blank group, and OD_c_ signifies the value of the control group

Live/dead cell staining was performed using a Calcein-AM/PI kit. The staining working solution was prepared by mixing 1 μL of Calcein-AM, 3 μL of 1.5 mM PI, and 1 mL of 1× assay buffer. MC3T3-E1 and L929 cells were treated in accordance with the MTT assay, washed with assay buffer after 24 h incubation, stained with the working solution for 15 min at 37°C in the dark, and imaged via laser confocal fluorescence microscopy.

**Figure S1.** Magnified view of the octahedral structure of [MnCl_3_]^-^ and [CdCl_3_]^-^. Σ denotes the sum of deviations from 90 degrees.

**Figure S2.** a) The image of DMAA-MnCl_3_ crystals emitting light under 365 nm ultraviolet light. b,c) The PLQY and emission decay curves of DMAA-MnCl_3_ crystals.

**Figure S3.** a) Emission curves of DMAA-MnCl_3_ at 20-120℃ at a 345 nm excitation wavelength. b) Emission curves of the DMAA-MnCl_3_ crystal and compressed state at a 345 nm excitation wavelength.

**Figure S4.** PLE spectral testing and the corresponding emission curves of DMAA-MnCl_3_ crystals at multiple excitation wavelengths.

**Figure S5.** Thermogravimetric analysis of DMAA-MnCl_3_ crystal.

**Figure S6.** a,b) Cell contraction comparison diagram of DMAA-MnCl_3_ and DMAA-CdCl_3_.

**Figure S7.** a) Lateral PFM phase images. b) PFM amplitude images. c) PFM topography images.

**Figure S8.** 3D surface plots of the elastic moduli of the DMAA-MnCl_3_ crystal in the room-temperature ferroelectric phase. a) Spatial orientation dependence of Young’s modulus. b) Spatial distribution of the maximum shear modulus. c) Spatial distribution of the minimum shear modulus.

**Figure S9.** Quasi-static *d*_33_ value of DMAA-MnCl_3_.

**Figure S10.** a,b) Measured stress-strain curves of the composite obtained by varying the content ratios of SEBS and SIS. c) Stress-strain curves of SEBS, SIS, SEBS/SIS.

**Figure S11.** Load-bearing test of SEBS/SIS.

**Figure S12**. a) Change in volume of the polymer before and after swelling. b) Comparison chart of swelling rates for blends with different weight ratios.

**Figure S13.** Actual images of SEBS/SIS before and after 700% tensile strain.

**Figure S14.** Loading-unloading compression curves of the SEBS/SIS.

**Figure S15.** Loading-unloading curves of the SEBS/SIS composite obtained from 2000 cyclic stretching tests.

**Figure S16**. a) SEM images and EDS Mapping of SEBS/SIS/DMAA-MnCl_3_ composite. b) Morphology image of DMAA-MnCl_3_ crystals in SEBS/SIS/DMAA-MnCl_3_ composite.

**Figure S17.** Emission curves of DMAA-MnCl_3_ crystal, SEBS/SIS/DMAA-MnCl_3_ composite at pristine, stretching, and compressed states at a 345 nm excitation wavelength.

**Figure S18**. XRD patterns of SEBS/SIS, DMAA-MnCl_3_, and SEBS/SIS/DMAA-MnCl_3_ composite.

**Figure S19**. Molecular interactions between the DMAA-MnCl_3_ and the SEBS/SIS matrix in the composites.

**Figure S20**. a) FI-TR spectra for SEBE/SIS and SEBE/SIS/DMAA-MnCl_3_. b) XPS spectra for DMAA-MnCl_3_ and SEBE/SIS/DMAA-MnCl_3_.

**Figure S21.** Loading-unloading compression curves of the SEBS/SIS/DMAA-MnCl_3_ composite.

**Figure S22.** Photographs of the SEBS/SIS/DMAA-MnCl_3_ composite at its pristine and stretching conditions under UV light irradiation.

**Figure S23.** Schematic diagram of piezoelectric sensor structure.

**Figure S24.** Piezoelectric response curves of different composite substrates and composites with different ferroelectric crystal contents under 30 N pressure.

**Figure S25**. a,b) Stress-strain curves and short-circuit current curves of the composites with different DMAA-MnCl_3_ contents (1, 5, 10, and 15 wt%).

**Figure S26**. a) Piezoelectric voltage outputs and b) GF values of the SEBS/SIS/DMAA-MnCl_3_ composite under 200%-500% strain.

**Figure S27**. a) Current response curves of the SEBS/SIS/DMAA-MnCl_3_ composite under different pressures (5, 10, 20, and 30 N). b) Current response curves of the SEBS/SIS/DMAA-MnCl_3_ composite under 200%-500% strain.

**Figure S28**. Piezoelectric voltage outputs of the SEBS/SIS/DMAA-MnCl_3_ composite under a 300% strain at -20, -30, -35, and -40℃.

**Figure S29.** GF values of the SEBS/SIS/DMAA-MnCl_3_ composite after being immersed in water for one day.

**Figure S30**. a, b) Confocal laser scanning microscopy images showing the viability of MC3T3-E1 and L929 cells. Scale bar: 100 μm. c, d) Viability of MC3T3-E1 and L929 cells assessed via the MTT assay after 24 h incubation.

**Figure S31**. The piezoelectric voltage output of SEBS/SIS/DMAA-MnCl_3_ composite over 2 months.

**Figure S32**. a-c) Mechanical properties, short-circuit current, and luminescent behavior of the SEBS/SIS/DMAA-MnCl_3_ composite after 10000 cycles.

**Figure S33**. a, b) Piezoelectric voltage outputs of the SEBS/SIS/DMAA-MnCl_3_ composite during 10000 consecutive cyclic compression and bending tests.

**Figure S34**. a) SNR, b) response/recovery time of the SEBS/SIS/DMAA-MnCl_3_ composite.

**Figure S35**. Voltage signals recorded during 90° finger bending, with the device fixed by BOPP transparent tape, PVC insulating tape, and medical breathable tape.

**Figure S36**. Three human subjects underwent one hour of prolonged exercise monitoring.

**Table S1.** Crystal data and structure refinements for DMAA-MnCl_3_.

| Compound | DMAA-MnCl_3_ | DMAA-MnCl_3_ |
| --- | --- | --- |
| Formula weight | 247.45 | 247.47 |
| Temperature/K | 293 | 383 |
| Crystal system | Orthorhombic | Orthorhombic |
| Space group | *Pna*2_1_ | *Pnma* |
| *a*/Å | 14.6296(5) | 14.8050(6) |
| *b*/Å | 10.7034(4) | 6.5257(3) |
| *c*/Å | 6.5236(2) | 10.8785(5) |
| *α*/° | 90 | 90 |
| *β*/° | 90 | 90 |
| *γ*/° | 90 | 90 |
| Volume/Å^3^ | 1021.51(6) | 1051.00(8) |
| *Z* | 4 | 2 |
| Density/g cm^3^ | 1.609 | 1.554 |
| *R*_1_ [*I* > 2*σ* (*I*)] | 0.0212 | 0.0456 |
| *wR*_2_ [*I* > 2*σ* (*I*)] | 0.0592 | 0.1429 |
| GOF | 1.092 | 1.123 |
| CSD number | 2541934 | 2541935 |

**Table S2**. Hydrogen bond geometry(Å, degree) at 293 K.

| D-H...A | d(D-H) | d(H...A) | d(D...A) | <(DHA) |
| --- | --- | --- | --- | --- |
| N(1) - H(1) - Cl(1) | 0.98 | 2.50 | 3.427(3) | 156.9 |
| ^1^1-X,1-Y,-1/2+Z; ^2^-1/2+X,3/2-Y,+Z; ^3^1-X,1-Y,1/2+Z | | | | |

**Table S3.** Bond lengths [Å] and bond angles [°] for DMAA-MnCl_3_ at 293 K.

| Bond lengths [Å] | | bond angles [°] | |
| --- | --- | --- | --- |
| Mn(1) - Cl(2)#1 | 2.5490(13) | Cl(2)#1 - Mn(1) - Cl(1) | 97.49(3) |
| Mn(1) - Cl(2) | 2.5548(13) | Cl(2) - Mn(1) - Cl(1)#1 | 97.87(3) |
| Mn(1) - Cl(1)#1 | 2.5809(11) | Cl(2)#1 - Mn(1) - Cl(1)#1 | 82.32(4) |
| Mn(1) - Cl(1) | 2.5769(11) | Cl(2) - Mn(1) - Cl(1) | 82.29(4) |
| Mn(1) - Cl(3) | 2.5457(12) | Cl(3)#1 - Mn(1) - Cl(2) | 95.02(3) |
| Mn(1) - Cl(3)#1 | 2.5376(11) | Cl(3)1 - Mn(1) - Cl(2)#1 | 83.98(4) |
|  |  | Cl(3) - Mn(1) - Cl(2) | 83.70(4) |
|  | | Cl(3)- Mn(1) - Cl(2)#1 | 97.30(3) |
|  | | Cl(3)#1 - Mn(1) - Cl(1)#1 | 84.88(4) |
|  | | Cl(3) - Mn(1) - Cl(1) | 84.79(4) |
|  | | Cl(3) - Mn(1) - Cl(1)#1 | 96.49(2) |
|  | | Cl(3)#1 - Mn(1) - Cl(1) | 93.85(2) |
| ^1^1-X,1-Y,-1/2+Z; ^2^1-X,1-Y,1/2+Z | | | |
